# Clinical and Molecular Epidemiology of Methicillin-Resistant *Staphylococcus aureus* in New Zealand: Rapid Emergence of Sequence Type 5 (ST5)-SCC*mec*-IV as the Dominant Community-Associated MRSA Clone

**DOI:** 10.1371/journal.pone.0062020

**Published:** 2013-04-25

**Authors:** Deborah A. Williamson, Sally A. Roberts, Stephen R. Ritchie, Geoffrey W. Coombs, John D. Fraser, Helen Heffernan

**Affiliations:** 1 Faculty of Medical and Health Sciences, University of Auckland, Auckland, New Zealand; 2 Department of Clinical Microbiology, Auckland District Health Board, Auckland, New Zealand; 3 Institute of Environmental Science and Research, Wellington, New Zealand; 4 PathWest Laboratory Medicine, Perth, Australia; 5 Curtin University, Perth, Australia; Rockefeller University, United States of America

## Abstract

The predominant community-associated MRSA strains vary between geographic settings, with ST8-IV USA300 being the commonest clone in North America, and the ST30-IV Southwest Pacific clone established as the dominant clone in New Zealand for the past two decades. Moreover, distinct epidemiological risk factors have been described for colonisation and/or infection with CA-MRSA strains, although these associations have not previously been characterized in New Zealand. Based on data from the annual New Zealand MRSA survey, we sought to describe the clinical and molecular epidemiology of MRSA in New Zealand. All non-duplicate clinical MRSA isolates from New Zealand diagnostic laboratories collected as part of the annual MRSA survey were included. Demographic data was collected for all patients, including age, gender, ethnicity, social deprivation index and hospitalization history. MRSA was isolated from clinical specimens from 3,323 patients during the 2005 to 2011 annual surveys. There were marked ethnic differences, with MRSA isolation rates significantly higher in Māori and Pacific Peoples. Over the study period, there was a significant increase in CA-MRSA, and a previously unidentified PVL-negative ST5-IV *spa* t002 clone replaced the PVL-positive ST30-IV Southwest Pacific clone as the dominant CA-MRSA clone. Of particular concern was the finding of several successful and virulent MRSA clones from other geographic settings, including ST93-IV (Queensland CA-MRSA), ST8-IV (USA300) and ST772-V (Bengal Bay MRSA). Ongoing molecular surveillance is essential to prevent these MRSA strains becoming endemic in the New Zealand healthcare setting.

## Introduction

The global emergence of community-associated methicillin-resistant *Staphylococcus aureus* (CA-MRSA) over the past two decades has been well described [1]. Of note, the predominant CA-MRSA vary between geographic settings, with USA300 the overwhelmingly dominant CA-MRSA clone in North America, and the ST93 Queensland CA-MRSA clone emerging as the most common CA-MRSA clone in Australia [2], [3]. In particular, the epidemic spread of the USA300 clone throughout North America has resulted in considerable morbidity, with several studies describing increasing rates of emergency department attendances and hospitalizations for CA-MRSA-associated skin and soft tissue infections (SSTI) [4], [5].

Initial reports of CA-MRSA strains described the emergence of these strains in patients with a lack of traditional epidemiological risk factors for MRSA infection and/or colonization [6]. In particular, patients with CA-MRSA infections were younger, had minimal comorbid illness and lacked prior healthcare contact compared to those patients with infections due to healthcare-associated MRSA (HCA-MRSA) strains [7], [8]. Furthermore, distinct socio-demographic associations have been described for CA-MRSA infection, including ethnicity, socioeconomic deprivation and incarceration [1]. Recently however, as the prevalence of CA-MRSA strains has increased, the epidemiological distinction between these strains and HCA-MRSA strains has become less well defined, with numerous reports of nosocomial outbreaks of infections due to CA-MRSA strains [9], [10].

In New Zealand, the ST30 South West Pacific (SWP) clone has been the predominant CA-MRSA for the past two decades [11]. Similar to prevalent CA-MRSA strains in other settings, ST30 SWP is characterized by carriage of the type IV SCC*mec* element, and of the *lukF-PV* and *lukS-PV* genes [12]. However, recent surveillance data suggest that an ST5-SCC*mec* IV (ST5-IV) CA-MRSA clone may be emerging as a more common cause of CA-MRSA infection in New Zealand [13]. To date however, relatively little is known about the distinct epidemiological associations for CA-MRSA infection in New Zealand, although a previous study suggested that infection with CA-MRSA strains may be more common in Māori (Indigenous New Zealander) and Pacific Island populations (Pacific Peoples) [14]. Moreover, the comparative burden and epidemiology of CA-MRSA and HCA-MRSA infections has not previously been described in our geographic setting.

In this context, we sought to (i) describe the clinical and molecular epidemiology of MRSA in New Zealand, and (ii) to describe the emergence of a previously unidentified ST5-IV MRSA strain as the predominant CA-MRSA clone in New Zealand.

This work was presented, in part, at the 52^nd^ Interscience Conference on Antimicrobial Agents and Chemotherapy, 2012 in San Francisco.

## Methods

### Setting, Patients and Data Collection

New Zealand is an island nation located in the South West Pacific, with a resident population of approximately 4.4 million. The population is ethnically diverse, consisting of 67% European, 15% Māori, 10% Asian, 7% Pacific Peoples and 1% of other ethnicities [15].

In New Zealand, a national survey of MRSA isolates is performed in August of each year. All New Zealand community and hospital laboratories refer consecutive, non-duplicate MRSA isolates to the Institute of Environmental Science and Research (ESR), Wellington, New Zealand for molecular typing. As part of this survey, basic patient data is collected, including age, gender and specimen type (clinical vs. screening isolate). We analyzed patient data from each year of the survey between 2005 and 2011. In order to minimize any influence that changes in screening practices may have had on MRSA isolation rates, demographic analysis was restricted to those patients who had a clinical isolate sent for testing. Using hospital admission data recorded by the New Zealand Ministry of Health, the following additional demographic information was extracted about each patient: ethnicity, number of previous hospitalizations in the preceding year, and socioeconomic status based on the New Zealand deprivation index (NZDep). The NZDep score is an area-based measure of deprivation derived from census data, and is based on the following variables of deprivation: household income, household ownership, household occupancy, employment and education levels, access to independent transportation and access to telecommunications. It is expressed as a score between one and ten, a score of ten representing the most deprived neighborhoods [16]. For analysis, ethnicity was grouped into four major ethnic groupings: European, Māori, Pacific Peoples and Asian/other ethnicities. When MRSA isolation was associated with a hospital attendance or admission, all hospital discharge diagnoses related to that event were obtained, coded using the *International Classification of Diseases, Tenth Edition, Clinical Modification* (*ICD-10-CM*) codes [17].

Cases were described as community-associated (CA-MRSA) if MRSA was isolated from a patient within 48 hours of hospital admission and/or who: (i) had no history of hospitalization or surgery in the preceding calendar year (ii) did not reside in a long-term care facility (LTCF) and (iii) did not have any prior or current *ICD-10-CM* discharge diagnoses relating to hemodialysis or implantable devices. Conversely, cases were described as healthcare-associated (HCA-MRSA) if one or more of these risk factors were documented. Similar to previously described methodology [18], we further described healthcare-associated cases as hospital-onset (HCA-HO) or community-onset (HCA-CO) depending on whether the specimen was taken >48 hours or ≤48 hours, respectively, following hospital admission.

### Isolate Collection and Analysis

Identification of *S. aureus* isolates and determination of methicillin resistance was carried out by individual laboratories prior to sending specimens to ESR. Prior to 2009, bacteriophage typing was the routine method used to type MRSA at ESR, as previously described [19]. From 2009 onwards, molecular typing was performed by PCR amplification and sequencing of the polymorphic X region of the staphylococcal protein A (*spa*) gene, using previously described methods [20]. An internal validation study was conducted in 2009 to ensure concordance between the two typing methods (data not shown). *spa* sequences were analysed using Ridom StaphType software, Version 2.0.3 (Ridom GmbH, Würzburg, Germany). Clustering of clonal complexes of related *spa* types (Spa-CCs) was performed using the based upon repeat pattern (BURP) algorithm of the Ridom software [21]. In addition to phage and *spa* typing, pulsed-field gel electrophoresis (PFGE) and multi-locus sequence typing (MLST) were used to further define and characterize MRSA strains. PFGE and MLST were performed as previously described [22], [23]. SCC*mec* typing and PCR amplification for detection of *lukF-PV* and *lukS-PV* genes was performed on representative isolates from each inferred MLST clonal complex, using previously described methods [24], [25].

### Statistics and Rates

Period-prevalence rates of MRSA isolation from clinical specimens were calculated per 100,000 population, and were stratified according to age and ethnicity. Population denominator data were obtained from Statistics New Zealand (http://www.stats.govt.nz). Categorical variables were compared using either the χ^2^ or Fisher’s exact test. Non-parametric data were analysed using the Mann-Whitney *U* test. Odds ratios were calculated with 95% confidence intervals. A regression model was used to determine changes in period-prevalence rates over time. A two-tailed *P* value of <0.05 was considered significant, and all statistical analysis was performed using SPSS software (Version 19).

### Ethics

The Multi-Region Ethics Committee, New Zealand granted ethical approval for this study. As this was an observational study using de-identified patient information, the Ethics Committee waived the need for written informed consent.

## Results

### Patients and Period-prevalence Rates of MRSA Isolations

**Figure 1:**
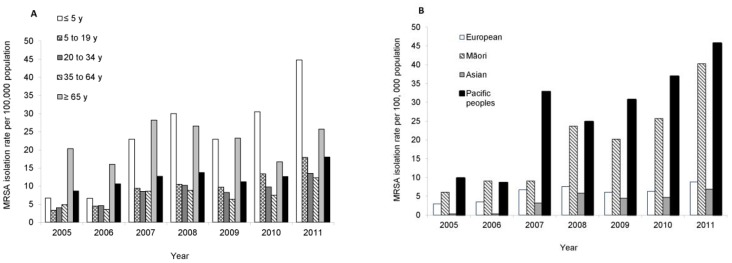
Methicillin-resistant *Staphylococcus aureus* (MRSA) period-prevalence rates for clinical isolates in New Zealand, 2005–2011, stratified by (A) age, and (B) ethnicity.

MRSA was isolated from clinical specimens from 3,323 patients during the 2005 to 2011 annual surveys. The overall period-prevalence rate of clinical MRSA isolation increased significantly from 8.6 to 18.0 per 100,000 population (*P*<0.001; Figure 1A). When stratified by age, the highest period-prevalence rates were in the under five and over 65 year age groups (Figure 1A). Full demographic information, including ethnicity was available for 2878 patients. When stratified by ethnicity, period-prevalence rates of MRSA isolations were consistently higher in Māori and Pacific Peoples (Figure 1B).

### Molecular Epidemiology of MRSA Strains and Emergence of ST5-IV Clone as the Dominant CA-MRSA Clone

Of the 3,323 isolates, 1569 (47.2%) were typed using phage typing and 1754 (52.8%) typed using *spa* typing. Ten major MRSA clonal complex (CC)/sequence types (ST) were identified over the study period (Table 1). The seven most frequent MRSA clones identified, accounting for 85.2% of all clinical MRSA, were ST30-IV (24.8%), ST5-IV (20.4%), ST22-IV (18.8%), ST1-IV (10.2%), ST8-IV (5.9%), ST93-IV (3.3%) and ST239-III (1.8%). Of the 3,323 MRSA isolates, 460 (13.9%) were unable to be assigned a clonal complex or sequence type by phage typing (278 isolates) or *spa* typing (182 isolates) (Table 1).

**Table 1:** Molecular epidemiology of methicillin-resistant *Staphylococcus aureus* isolates referred to the Institute of Environmental Science and Research for New Zealand annual MRSA surveys between 2005–2011.

Predicted clonal complex/sequence type	Number analysed by bacteriophage typing between 2005–2008	Number analysed by *spa* typing between 2009–2011	*spa* types (n)
**ST30 (n = 825)**	484	341	t019 (309); t975(5); t1347(5); t138(4); t122(3); t1752(3); t021(2); t779(2); t1133(1); t1273(1); t2895(1); t3723(1); t4341(1); t4672(1); t5994(1); t6653(1)
**ST5 (n = 678)**	148	530	t002(461); t045(23); t548(10); t062(7); t306(7); t010(4); t105(3); t088(2); t1781(2); t5677(2); t009(1); t214(1); t242(1); t311(1); t1154(1); t4323(1); t4865(1); t5213(1); t6787(1)
**ST22 (n = 625)**	404	221	t032(153); t1401(14); t022(12); t1214(5); t852(4); t5501(4); t005(3); t379(3); t5538(3); t718(2); t790(2); t5785(2); t7428(2); t557(1); t578(1); t688(1); t749(1); t906(1); t1370(1); t1415(1); t1467(1); t3612(1); t5836(1); t605(1); t6648(1)
**ST1 (n = 340)**	129	211	t127(178); t701(11); t267(5); t386(3); t591(3); t7136(3); t359(1); t521(1); t559(1); t693(1); t1418(1); t5100(1); t5736(1); t5837(1)
**ST8 (n = 196)**	58	138	t008(118); t024(10); t2849(2); t6229(2); t711(1); t1610(1); t1627(1); t1882(1); t2558(1); t4919(1)
**ST93 (n = 110)**	10	100	t3949(66); t202(30); t1819(1); t4178(1); t6487(1); t7328(1)
**ST239 (n = 61)**	47	14	t037(10); t631(1); t4150(2); t4866(1)
**ST772 (n = 14)**	2	12	t657(10); t345 (1); t5310 (1)
**ST36 (n = 11)**	9	2	t018(2)
**CC398 (n = 3)**	–	3	t034(2); t011(1)
**Not determined (n = 460)**	278	182	t1853(24); t976(16); t375(14); t189(11); t437(9); t324(8); t282(6); t148(4); t065(3), t179(3); t186(3); t216(3); t316(3); t1265(3); t1767(3); t2143(3); t019(2); t021(2); t084(2); t121(2); t878(2); t1781(2); t9257(2); t8493(2); t005(1); t026(1); t040(1); t044(1); t064(1); t088(1); t089(1); t127(1); t237(1); t304(1); t442(1); t586(1); t638(1); t688(1); t690(1); t771(1); t786(1); t791(1); t985(1); t1028(1); t1126(1); t1309(1); t1839(1); t1849(1); t1895(1); t2062(1); t2078(1); t2085(1); t2321(1); t2815(1); t3979(1); t4359(1); t4448(1); t4467(1); t5110(1); t5181(1); t5190(1); t5191(1); t5562(1); t5782(1); t5784(1); t6256(1); t6681(1); t7211(1); t7334(1); t8349(1); t9258(1); t9269(1); t9508(1)
**Total (n = 3323)**	**1569**	**1754**	**–**

**Figure 2:**
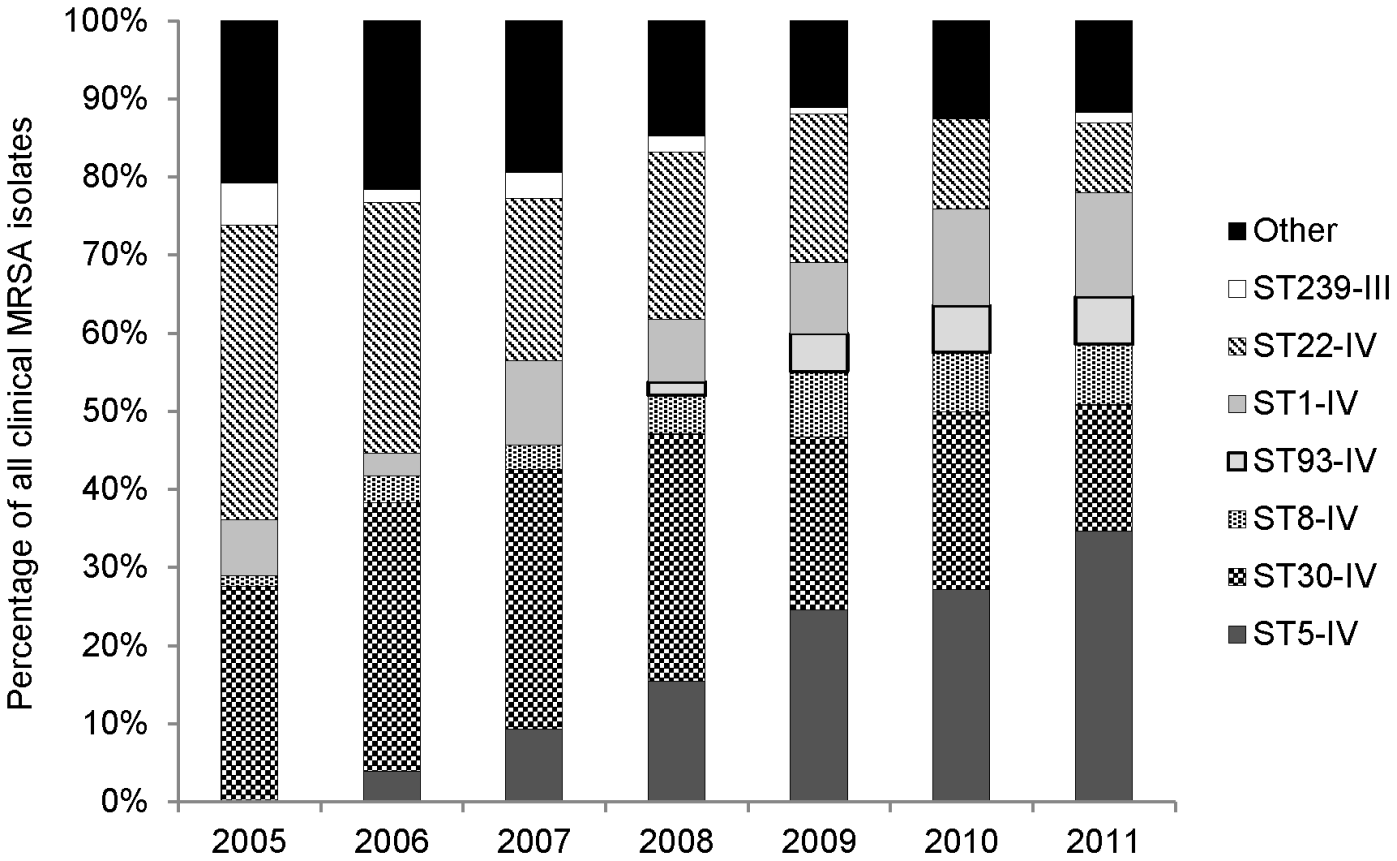
Relative proportions of methicillin-resistant *Staphylococcus aureus* (MRSA) clones circulating in New Zealand, 2005–2011.

An ST5-IV MRSA clone was identified for the first time in the 2005 survey, and rapidly became the predominant CA-MRSA clone in New Zealand. Representative isolates from this clone were negative for the *lukF-PV* and *lukS-PV* genes. The relative proportions of the seven predominant MRSA clones isolated from clinical specimens between 2005–2011 are shown in Figure 2. Notably, the proportion of ST5-IV increased significantly over the study period, from 0.3% in 2005 to 34.7% in 2011 (*P*<0.001), and the proportions of ST30-IV and ST22-IV decreased significantly from 28.1 to 16.2% (*P*<0.001) and 37.8 to 8.9% (*P*<0.001), respectively.

There was an age-related distribution of the seven commonest MRSA clones, with ST22-IV isolated from older patients (median age 79 years, interquartile range [IQR] 62–86 years), followed by ST239-III (median 69 years, IQR 48–79 years), ST8-IV (median 37 years, IQR 18–61 years), ST30-IV (median 27 years, IQR 14–43 years), ST1-IV (median 26 years, IQR 7–53 years) and ST5-IV (median 17 years, IQR 4–42 years).

### Isolation of other Emerging MRSA Strains in New Zealand

Fourteen patients with ST772-V MRSA (Bengal Bay MRSA) were identified over the study period (2 patients in 2008; 4 in 2009; 2 in 2010 and 6 in 2011). Ten of these patients were of Indian ethnicity. The *spa* types associated with ST772-V were t657 (10 isolates), t345 (one isolate) and t5310 (one isolate).

In addition, three patients with CA-MRSA isolates belonging to CC398 were identified in 2011. The associated *spa* types were t034 (2 isolates) and t011.

### Community vs. Healthcare-associated MRSA in New Zealand

**Figure 3:**
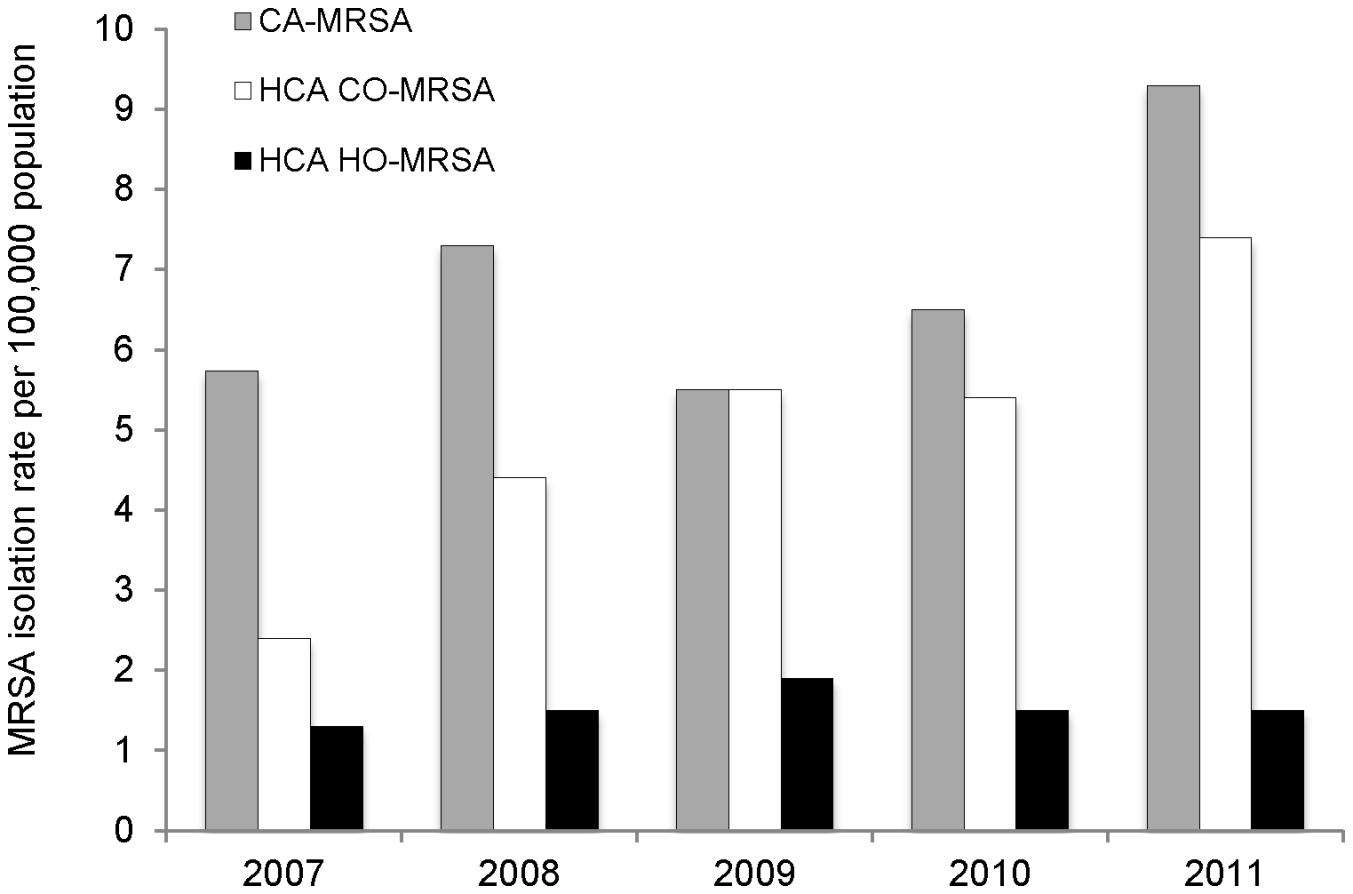
Period-prevalence rates of community-associated methicillin-resistant *Staphylococcus aureus* (CA-MRSA), healthcare-associated community-onset MRSA (HCA-CO MRSA) and healthcare-associated hospital-onset (HCA-HO MRSA) in New Zealand, 2005–2011.

The period-prevalence rates of CA-MRSA and HCA-MRSA infections are shown in Figure 3. The period-prevalence rate of CA-MRSA increased significantly over the study period from 5.7 to 9.3 per 100,000 population (*P*<0.001). In addition, the period-prevalence rate of HCA-CO MRSA increased from 2.4 to 7.4 per 100,000 population (*P*<0.001). In contrast, the period-prevalence of HCA-HO MRSA did not change significantly over the study period (Figure 3).

In comparison to patients with HCA-MRSA, patients with CA-MRSA were significantly younger, more likely to be Māori or Pacific Peoples, and were more likely to reside in a deprived neighborhood (Table 2). In addition, there were distinct associations between specific MRSA clones and their isolation in either the community or healthcare setting. ST30-IV, ST5-IV, and ST93-IV were significantly more likely to be isolated from patients in the community, whereas ST22-IV and ST239-III were more likely to be isolated from patients in a healthcare setting (Table 2).

**Table 2:** Sociodemographic and molecular epidemiological associations of community-associated MRSA (CA-MRSA) and healthcare-associated MRSA (HCA-MRSA) in New Zealand, 2005–2011.

Category	Subcategory	CA-MRSA (n = 1346)	HCA CO-MRSA (n = 1132)	HCA-HO-MRSA (n = 400)	CA-MRSA vs. HCA CO-MRSA		CA-MRSA vs. HCA HO-MRSA	
					*P*	OR (95% CI)	*P*	OR (95% CI)
Age, median, years (IQR)		27 (10–47)	50 (19–78)	64 (29–80)	<0.001^a^	–	<0.001^a^	–
Gender	Male	692 (51.4)	590 (52.1)	230 (57.5)	0.75	0.97 (0.83–1.14)	0.032	0.78 (0.62–0.99)
	Female	654 (49.6)	542 (47.9)	170 (42.5)	–	–	–	–
Ethnicity	European	470 (34.9)	559 (49.4)	230 (57.5)	0.001	0.8 (0.6–0.9)	<0.001	0.4 (0.3–0.5)
	Maori	466 (34.7)	332 (29.3)	97 (24.2)	0.005	1.3 (1.1–1.5)	<0.001	1.7 (1.3–2.2)
	Pacific Peoples	345 (25.6)	203 (17.9)	58 (14.5)	<0.001	1.6 (1.3–1.9)	<0.001	2.1 (1.5–2.8)
	Other	65 (4.8)	38 (3.4)	15 (3.8)	0.07	1.5 (0.9–2.2)	0.36	1.3 (0.7–2.4)
NZDep score	1–3 (low deprivation)	158 (11.7)	171 (15.1)	70 (17.5)	0.014	0.7 (0.6–0.9)	0.003	0.6 (0.5–0.9)
	4–7 (medium deprivation)	362 (26.9)	311 (27.5)	129 (32.2)	0.75	0.9 (0.8–1.2)	0.036	0.8 (0.6–1.0)
	8–10 (high deprivation)	826 (61.4)	650 (57.4)	201 (50.3)	0.046	1.2 (1.0–1.4)	<0.001	1.6 (1.2–2.0)
MRSA clone	ST30-IV	438 (32.5)	232 (20.5)	50 (12.5)	<0.001	2.2 (1.8–2.7)	<0.001	3.4 (2.4–4.7)
	ST5-IV	335 (24.9)	218 (19.3)	48 (12.0)	0.001	1.4 (1.1–1.7)	<0.001	2.4 (1.7–3.4)
	ST22-IV	87 (6.5)	282 (24.9)	154 (38.5)	<0.001	0.25 (0.2–0.3)	<0.001	0.1 (0.001–0.2)
	ST1-IV	161 (11.9)	106 (9.4)	38 (9.5)	0.04	1.3 91.0–1.7)	0.17	1.3 (0.9–1.9)
	ST8-IV	80 (5.9)	66 (5.8)	23 (5.8)	0.93	1.0 (0.7–1.5)	1.0	1.0 (0.6–1.7)
	ST93-IV	64 (4.8)	33 (2.9)	9 (2.2)	0.02	1.7 (1.1–2.7)	0.03	2.2 (1.6–4.7)
	ST239-III	5 (0.4)	22 (1.9)	29 (7.3)	<0.001	0.2 (0.1–0.5)	<0.001	0.05 (0.02–0.1)

Note: Data are number (%) of patients unless stated otherwise.

a Mann-Whitney *U* test.

Abbreviations: OR, odds ratio; CI, confidence interval; IQR, interquartile range; ST, sequence type, NZDep, New Zealand Deprivation Index score; MRSA, methicillin-resistant *Staphylococcus aureus*.

## Discussion

To our knowledge, this represents the first study to systematically assess the clinical and molecular epidemiology of MRSA from New Zealand. We analyzed seven years of data from our national MRSA survey, and found that, although rates of MRSA isolation were generally low in comparison to other developed countries, there were notable demographic and epidemiological findings.

Most notably, an ST5-IV clone rapidly emerged over the study period and displaced ST30-IV as the dominant CA-MRSA clone. Interestingly, unlike many other CA-MRSA clones, including ST30-IV, this ST5-IV clone characteristically does not harbor the *lukF-PV* and *lukS-PV* genes. MRSA strains belonging to ST5 represent a large and diverse group, and are geographically widespread [26], [27]. The origin of this recently emerged ST5-IV strain in our region is unclear, but based on studies from other settings it seems probable that this clone emerged from a locally circulating ST5 methicillin-susceptible *S. aureus* (MSSA) strain which acquired the small, mobile SCC*mec*-IV element [27]. Future work should attempt to characterize the possible clinical and molecular reasons for the successful emergence of this clone in New Zealand.

In addition, we observed a significant increase in the burden of CA-MRSA disease over the study period. In our setting, this was largely due to two CA-MRSA clones, ST30-IV and ST5-IV. The reasons for the marked increase in CA-MRSA over the study period are unclear, but may, in part, reflect the increasing incidence of SSTI in New Zealand over the past decade [28], [29]. Of note, the population groups with the highest burden of SSTI in New Zealand, namely Māori and Pacific Peoples, are the same population groups that carry the greatest burden of CA-MRSA disease. A strong association between CA-MRSA disease and distinct indigenous groups in other settings has been previously described, including Aboriginal Australians [30], and American Indians [1]. The reasons for this association are multifactorial, but are likely to involve a combination of poor hygiene, nutrition and domestic crowding [28]–[30]. Further research should attempt to understand the interaction of host and sociodemographic factors that predispose to CA-MRSA infection in these groups.

We also observed a three-fold increase in the burden of community-associated MRSA disease in patients with prior healthcare exposure (HCA-CO MRSA). The reasons for this significant increase are uncertain, but it seems probable that a number of factors may have contributed, including an increase in the community reservoir of MRSA and changes in the delivery of healthcare, such that many medical treatments and procedures are now undertaken in a community setting. As such, the clinical and molecular epidemiology of HCA-CO MRSA disease may share both community and hospital characteristics. In our data, this was reflected by the specific MRSA clones causing HCA-CO disease; it was notable that ST5-IV and ST30-IV, both clones which are predominantly associated with CA-MRSA, contributed to approximately 40% of HCA-CO MRSA isolates. The infiltration of CA-MRSA clones into the healthcare setting has been recently described, and it has been suggested that CA-MRSA strains may ultimately replace traditional HCA-MRSA strains in the healthcare environment [9]. However, despite the increase in HCA-CO MRSA, we did not observe any increase in HCA-HO MRSA over the study period. This may, in part, be due to local initiatives over the past several years designed to prevent healthcare-associated infections, in particular a national campaign to improve hand hygiene compliance in acute care settings [31]. Future efforts should be directed towards reducing the rate of HCA-HO MRSA even further, in addition to addressing the increasing burden of HCA-CO MRSA.

Interestingly, we also observed the emergence of several clones associated with CA-MRSA disease in other geographic settings, most notably the ST8-IV USA300 clone from North America and the ST93-IV Queensland clone from Australia. Although we did not have specific epidemiological information relating to overseas travel, it seems probable that emergence of these clones in New Zealand was due to sporadic introduction and “spillover” from other settings with ensuing community spread, rather than local emergence of these clones. Of concern was the observation that the USA300 clone accounted for approximately 6% of HCA-HO MRSA isolations over the study period. This successful clone is notable for its apparent transmissibility and ability to harbor several virulence factors [1]. Of further concern was our finding of fourteen MRSA isolates belonging to ST772-V. ST772-V MRSA, also dubbed the “Bengal Bay clone”, is noted for its resistance to multiple antibiotic classes and its ability to harbor several virulence factors including the *lukF-PV* and *lukS-PV* genes and the enterotoxin gene cluster (*egc*) [32]. An epidemiological link with travel to the Indian subcontinent has previously been described for patients from whom ST772 MRSA has been isolated [32]. Although we did not have information related to overseas travel, it is notable that of the fourteen patients with ST772 MRSA in New Zealand, ten (71%) were of Indian ethnicity.

There were a number of limitations with our study. Most notably, we did not have information relating to clinical syndromes associated with specific MRSA clones. Future prospective studies should monitor the type and severity of disease caused by MRSA in our setting, particularly in light of the recent emergence of the *lukF-PV*/*lukS-PV-*negative ST5-IV clone. In addition, the data we analyzed was based on annual monthly “snapshot” surveys, rather than continuous surveillance. As such, we had no information on MRSA colonisation/infection from the time periods outside the annual surveys. Despite this limitation however, isolates and data were collected consistently each year, and from all diagnostic microbiology laboratories in New Zealand, thus providing a nationally representative profile of the epidemiology of MRSA. Finally, in this study, we focused exclusively on MRSA. In our setting however, *S. aureus* disease is predominantly due to MSSA strains, which frequently harbor the *lukF-PV*/*lukS-PV* genes [25]. Future studies should attempt to assess the clinical and molecular epidemiology of disease caused not only by MRSA, but also by MSSA.

In summary, we found notable temporal and sociodemographic differences in the epidemiology of MRSA in New Zealand. The increase in CA-MRSA was consistent with reports from other settings, and the burden of CA-MRSA was disproportionately carried by the Māori and Pacific Peoples ethnic groups. We identified several MRSA clones not previously known to circulate in New Zealand, including an ST5-IV clone that rapidly displaced ST30-IV as the dominant CA-MRSA clone. Of particular concern was the finding of several successful and virulent MRSA clones from other geographic settings, including ST93-IV (Queensland CA-MRSA), ST8-IV (USA300) and ST772-V (Bengal Bay MRSA). Ongoing molecular surveillance is essential to ensure early identification and tracking of such strains, and to prevent these MRSA strains becoming endemic in the New Zealand healthcare setting.
